# Complete remission following pembrolizumab in a man with mCRPC with both microsatellite instability and *BRCA2* mutation

**DOI:** 10.1093/oncolo/oyae156

**Published:** 2024-06-26

**Authors:** Casey Moore, Isabel Naraine, Tian Zhang

**Affiliations:** Division of Hematology and Oncology, Department of Internal Medicine, Simmons Comprehensive Cancer Center, UT Southwestern Medical Center, Dallas, TX 75390-8852, United States; Division of Hematology and Oncology, Department of Internal Medicine, Simmons Comprehensive Cancer Center, UT Southwestern Medical Center, Dallas, TX 75390-8852, United States; Division of Hematology and Oncology, Department of Internal Medicine, Simmons Comprehensive Cancer Center, UT Southwestern Medical Center, Dallas, TX 75390-8852, United States

**Keywords:** prostate cancer, metastatic hormone sensitive, androgen receptor, pembrolizumab, precision medicine, personalized treatment

## Abstract

Prostate cancer is one of the most prevalent malignancies in men. In the United States, 1 in 8 men will be diagnosed with prostate cancer in their lifetime. Specifically, studies have delved into male subgroups that present a heightened risk for prostate cancer. Despite such high prevalence, prostate cancer can be heterogeneous and carry complexities that manifest differently between individuals. Metastatic hormone-sensitive prostate cancer (mHSPC) often has an abbreviated, aggressive disease course, and can have varying presentations with different molecular profiles that determine response/resistance to the approved treatments targeting the androgen-receptor pathway (eg, enzalutamide, apalutamide, darolutamide, and abiraterone acetate). We present a case of mHSPC quickly progressing to mCRPC, found to have microsatellite instability in mCRPC and excellent response to pembrolizumab, which raises the critical issues of early molecular testing and treatments personalized for the individual patient.

## Teaser

Metastatic hormone-sensitive prostate cancer (mHSPC) often has an abbreviated, aggressive disease course. This article presents a case of mHSPC quickly progressing to mCRPC, found to have microsatellite instability in mCRPC and excellent response to pembrolizumab, raising the critical issues of early molecular testing and treatments personalized for the individual patient.

**Key Points**

- A small percentage of patients with metastatic prostate cancer have alterations in mismatch repair genes, high tumor mutational burden (TMB), along with concurrent mutations in homologous recombination repair (HRR) pathway genes.
- In patients with microsatellite instability (MSI-high) and mutations in additional targetable genes such as the HRR pathway, there is no clear therapy preference in checkpoint blockade and PARP inhibition.
- Patients with high TMB or MSI-high with concurrent mutations in HRR genes may benefit from immunotherapy prior to PARP inhibition.

## Introduction

Immunotherapy, particularly immune checkpoint blockade (ICB), has dramatically changed cancer treatment. However, most patients with metastatic castration-resistant prostate cancer (mCRPC) do not respond to checkpoint blockade either targeting CTLA4 or the PD1/PDL1 axis. While some individuals with mCRPC can have durable responses to ipilimumab,^1^ phase 3 clinical trials of ipilimumab versus placebo in either chemotherapy naïve or docetaxel/radiotherapy pretreated patients have shown no improvement in overall survival.^2,3^ While pembrolizumab has been observed to have a prostate specific antigen (PSA) response in patients with mCRPC,^4^ and a phase 2 trial of pembrolizumab with enzalutamide showed early signal,^5^ phase 3 trials adding pembrolizumab to standard treatments did not prove effective across the population.^6-8^ However, early phase 2 studies showed a signal for the combination of ipilimumab/nivolumab in patients with mutations in the DNA damage repair (DDR) pathway, but none of the tumors had microsatellite instability (MSI) and TMB was less than 8 Muts/Mb.^9,10^ Retrospective analysis of patients with metastatic prostate cancer treated with pembrolizumab who have alterations in mismatch repair pathway has shown higher response rates.^11^

We report a case in which a patient with rapidly progressive metastatic castrate-resistant prostate cancer (mCRPC) was found to have an *MSH6* mutation with microsatellite instability (MSI) and notably high tumor mutational burden (TMB) 109 mut/Mb, who had a complete response to pembrolizumab. Coincidentally, he also had copy number loss of *BRCA2*. Notable anecdotal evidence like this suggest that ICB should be considered prior to PARP inhibitors for disease control in patients with mCRPC with high TMB or MSI-high.

## Case

**Figure 1.**
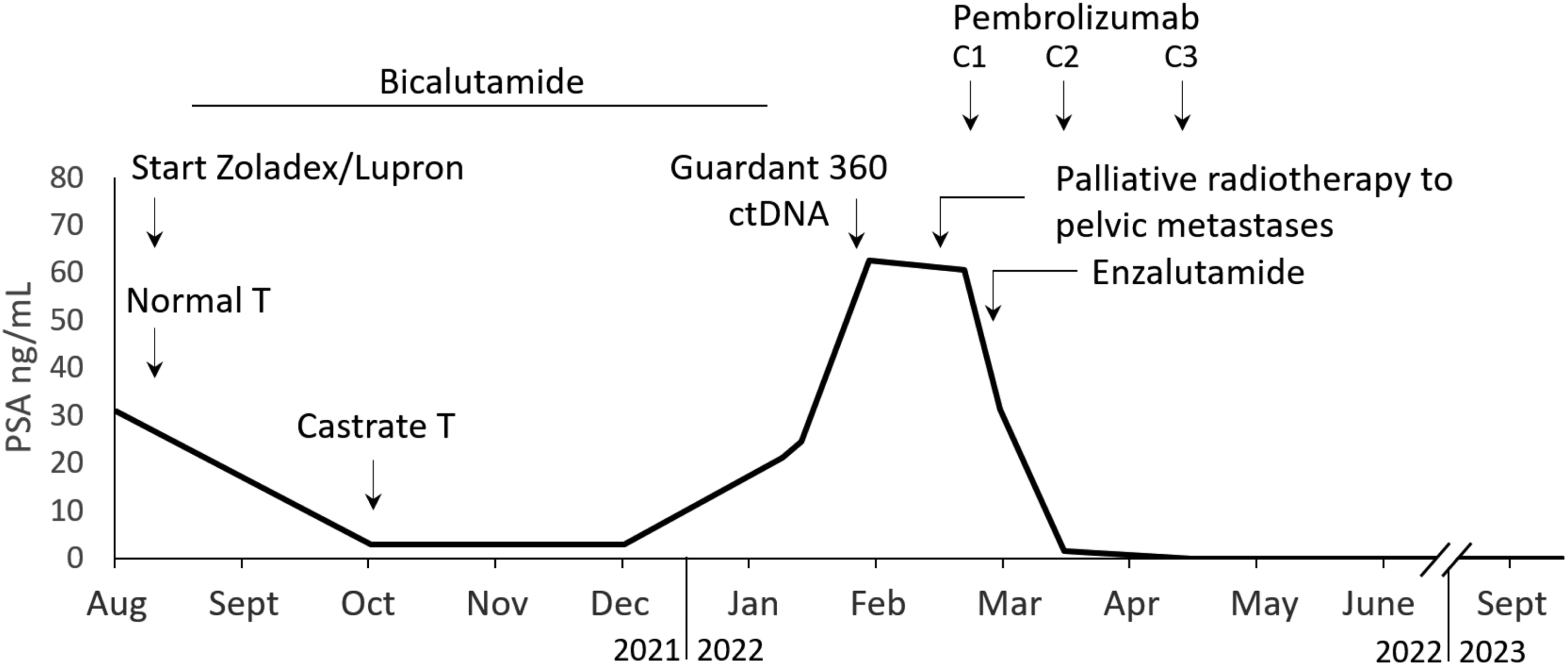
Serum prostate specific antigen (PSA) over time.

An 81–year-old man presented in August 2021 with increased PSA 30.8 ng/mL, normal testosterone, and bone-metastatic HSPC at baseline, with a prior history of dermatomyositis on weekly methotrexate. He was initially started on zoladex/bicalutamide, with initial PSA response and PSA nadir 2.9 ng/mL (Figure 1). He underwent an initial biopsy of a bony metastasis showing prostate adenocarcinoma without neuroendocrine differentiation. IHC was negative for Ck7, Ck20, TTF-1 and p63, positive for AE1/AE3, racemase, PSA and NKX3.1.

He presented to our cancer center 5 months after starting ADT and bicalutamide. Upon presentation, the patient had high-volume metastases, PSA was rapidly rising, he was symptomatic with significant bone pain, and had multiple new lesions on bone scan without visceral or lymph node metastasis. He received palliative radiotherapy to a total dose of 20 Gy in 5 fractions to the pelvic bone metastases which improved his pain. Upon presentation, his circulating tumor DNA (ctDNA) was sequenced using the Guardant 360 assay and notably showed microsatellite instability (MSI-high) and *MSH6* S702* loss; *BRCA2* copy number loss/loss of heterozygosity along with 2 *BRCA2* synonymous mutations (V1438V and V821V); several *AR* mutations: W742C, W742L, M896V, P893S, and S889G; *SMAD4* mutations: R361H, L540P, and RB1 R661W; and significantly elevated TMB 109 mut/Mb. He had underlying dermatomyositis well controlled on weekly methotrexate. Given the MSI-high status and baseline immune suppression, he stopped methotrexate and began cycle 1 of pembrolizumab and enzalutamide when his PSA was 60.55 ng/mL. He achieved 50% PSA decline after the first cycle, then over 95% PSA decline after cycle 2 and after cycle 3, PSA was undetectable (Figure 1).

During and after treatment with pembrolizumab, the patient did not have any symptoms consistent with dermatomyositis flare. However, 1 day after cycle 3 pembrolizumab treatment, the patient developed severe headaches with concern for hypophysitis versus dural inflammation. CT imaging showed no radiographic abnormalities suggesting hypophysitis. The headaches improved with prednisone 40 mg daily tapered over 2 months. A few days after the headaches, the patient developed a rash evaluated by dermatology to be a lichenoid drug eruption secondary to pembrolizumab. It was treated with clobetasol cream and required extending the prednisone taper mentioned above, with improvement over 2 months.

**Figure 2.**
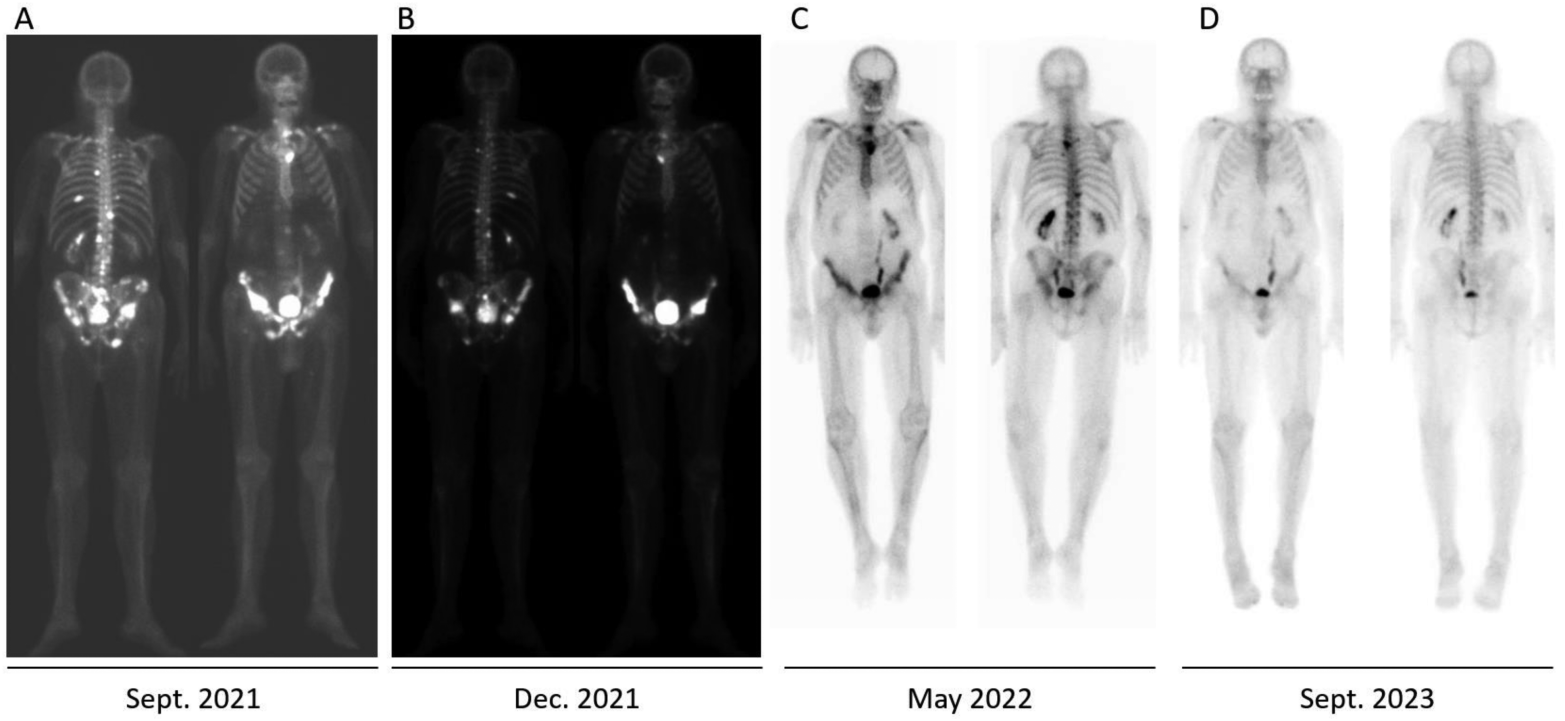
Sequential bone scans at the time of presentation (A), detection of MSI-high status (B), after 3 cycles on treatment (C), and approximately 16 months after treatment (D) with undetectable PSA.

Given the undetectable PSA and concern for ongoing immune-related toxicities, pembrolizumab was permanently discontinued. After pembrolizumab was stopped, the patient remained on ADT and enzalutamide for another 14 months. Enzalutamide was discontinued in June 2023. As of the last follow-up in March 2024, he has had a sustained response with undetectable PSA and improved bone metastases on bone scan (Figure 2).

## Discussion

### Genetic alterations in prostate cancer

Genomic studies in the last decade have identified several molecular pathways often mutated in prostate cancer which have both prognostic and therapeutic significance. These pathways involve DDR mechanisms, specifically homologous recombination repair (HRR) and mismatch repair (MMR). Specifically, men with metastatic prostate cancer are at higher risk of having germline mutations in these pathways, with up to 11.8% having pathogenic mutations compared to only 2.4% of all men with prostate cancer (including localized disease).^12^ Studies have shown between 15-23% of patients with mCRPC have somatic or germline mutations in HRR genes including *BRCA2*, *BRCA1*, *ATM*, and *CDK12*.^13-15^ Furthermore, clinical studies show that mutations in DDR genes are more common in the tumors of patients who progressed from castrate sensitive to resistant and in tumors recurring in abdominal nodes, bone, or viscera.^16^

The mismatch repair pathway also promotes prostate cancer formation and progression as patients with Lynch syndrome harboring germline mutations in MMR genes are at increased risk. Previous investigations suggest that MSI-high status and mutations in MMR genes (*MLH1*, *MSH2*, *MSH6*, and *PMS2*) are detectable in around 3% of prostate cancers.^17,18^

### Targeted treatment of prostate cancer based on genetic profiling

There are now several FDA-approved targeted therapies available for patients with mCRPC with somatic or germline mutations in genes involved in HRR and MMR. Patients with metastatic prostate cancer are recommended to undergo genetic testing, as genetic results can significantly influence treatment choice and patient outcomes. Patients with HRR defects benefit from PARP inhibition. Recent clinical trials including PROfound and TRITON2 showed benefits in overall survival in patients with pathogenic mutations in either *BRCA1/2* or other HRR genes after treatment with olaparib or rucaparib, respectively, which led to subsequent approvals for treatment of mCRPC.^19,20^ Moreover, the data from PROPEL, TALAPRO-2, and MAGNITUDE showed progression-free survival for mCRPC prior to chemotherapy, and subsequently led to approvals of, respectively, olaparib with abiraterone acetate, talazoparib with enzalutamide, and niraparib with abiraterone acetate.^21-23^

After the positive results from multicenter phase 2 trials Keynote 158 (NCT02628067), KEYNOTE-164 (NCT02460198), and KEYNOTE-051 (NCT02332668), the FDA approved pembrolizumab for the treatment of all patients with MSI-high and TMB high solid tumors who have progressed on previous treatment and have no satisfactory alternative options. While there were few patients with prostate cancer included in the original trials, subsequent studies of patients with mCRPC with MSI-high disease show a favorable response in patients with prostate cancer and MSI-high disease with or without high TMB.^11,17^ Several recent case reports have shown complete responses to pembrolizumab in pretreated patients with mCRPC with MSI-high. In one case, the patient had MSI-high status and neuroendocrine differentiation with sustained clinical response at 14 months.^24^ Another had TMB of 61 mutations/Mb and immunohistochemical loss of MSH2 and MSH6 with a complete response to pembrolizumab sustained at 18 months.^25^ Our patient had an equally dramatic response to only 3 cycles of pembrolizumab. It is important to note that both our patient and those referenced had tumors with TMB > 10 mut/Mb and MSI-high status. Therefore, these dramatic results may not hold true for the subset of patients with MSI-high but TMB < 10 mut/Mb.

Finally, it is important to note that the patient was treated with enzalutamide concurrently with pembrolizumab. We believe the dramatic treatment response was due mostly to the pembrolizumab given the high TMB and sustained disease response after discontinuation of both enzalutamide and pembrolizumab.

### Sequencing of treatments in mCRPC based on genetic signatures

The patient presented here had a mutation in *MSH6,* resulting in microsatellite instability and exceptionally high TMB, as well as copy number loss in *BRCA2*. He was therefore a candidate for either PARP inhibition given his *BRCA2* loss, or pembrolizumab given MSI-high status and high TMB. This is not uncommon in patients with mCRPC with MSI-high disease. In a cohort of 14 patients with mCRPC with MSI-high disease, 29% had additional mutations in *BRCA1*, 21% in *BRCA2*, 14% in *ATM,* and 7% in *CDK12*^11^. Notably, the sequencing of PARP inhibitors or ICB has not been explored/evaluated. An interesting point to consider is which mutation is functioning as a driver mutation, and whether blocking PARP with an inhibitor in the setting of microsatellite instability would be as effective as checkpoint inhibition. In this particular setting, MSI-high status likely drove the *BRCA2* loss and determined the exquisite sensitivity to pembrolizumab. In a recent genomic profiling cohort study, 2 patients with *BRCA1/2* monoallelic mutations who were also MSI-high were not sensitive to PARP inhibition, suggesting the *BRCA1/2* mutation or loss may be carrier mutations rather than a driver when found concurrently to MSI-high status.^26^

Importantly, patients with MSI-high mCRPC seem highly susceptible to ICB; a multi-institutional case series of 9 patients with MSI-high mCRPC treated with pembrolizumab showed 4/9 (44%) achieved 50% PSA decline after treatment, including 3 patients with >99% decline. Of the 4 with PSA decline over 50%, 2 had pathogenic alterations in *BRCA1/2* like our patient.^11^ Given this response, *BRCA* mutations are postulated to be a biomarker of response to ICB in patients with MSI-high disease. These and our study suggest that in the few patients with co-occurring HRR mutations and MSI-high tumors, treatment with pembrolizumab can be highly successful, but further studies are needed to guide appropriate treatment sequencing in these rare circumstances. Understanding the hierarchy of importance between biomarkers involved in mCRPC is vital to sequencing effective treatments. In our patient’s case, where *BRCA2* monoallelic loss of heterozygosity was found with only synonymous *BRCA2* mutations, the *BRCA2* loss was likely a carrier of microsatellite instability and unlikely a driver molecular alteration. Attention should be paid on molecular reports for *BRCA*1/2 monoallelic versus biallelic loss.

## Conclusion

We present a unique case of a patient with initial mHSPC, who quickly progressed to mCRPC, presenting with 3 actionable genomic alterations to guide treatment selection: high TMB, MSI-high status, and *BRCA2* copy number loss. Coinciding HRR mutations are not infrequent among patients with microsatellite instability, and few cases suggest these patients may benefit from early ICB treatment and delaying PARP inhibitor treatment. The patient described here was treated successfully with only 3 cycles of pembrolizumab and has had a complete response with undetectable PSA now 21 months after his last cycle of pembrolizumab. This case suggests that patients with progressive metastatic prostate cancer should receive genomic analysis of their tumor or cfDNA, and if coinciding HRR and MMR mutations co-exist, immunotherapy should be used first prior to PARP inhibition, as other mutations may be bystanders. Prostate cancer is still evolving in targeted treatment approaches. In rare patients such as this one with mCRPC with MSI-high status, ICB should be preferred as the first treatment option.

## Data Availability

The data underlying this article will be shared on reasonable request to the corresponding author.
